# The Application of Knowledge-Based Clinical Decision Support Systems to Enhance Adherence to Evidence-Based Medicine in Chronic Disease

**DOI:** 10.1155/2023/8550905

**Published:** 2023-05-29

**Authors:** Marsa Gholamzadeh, Hamidreza Abtahi, Reza Safdari

**Affiliations:** ^1^Medical Informatics, Health Information Management and Medical Informatics Department, School of Allied Medical Sciences, Tehran University of Medical Sciences, Tehran, Iran; ^2^Thoracic Research Center, Imam Khomeini Hospital Complex, Tehran University of Medical Sciences, Tehran, Iran; ^3^Pulmonary and Critical Care Department, Thoracic Research Center, Imam Khomeini Hospital, Tehran University of Medical Sciences, Tehran, Iran; ^4^Health Information Management and Medical Informatics Department, School of Allied Medical Sciences, Tehran University of Medical Sciences, Tehran, Iran

## Abstract

Among the technology-based solutions, clinical decision support systems (CDSSs) have the ability to keep up with clinicians with the latest evidence in a smart way. Hence, the main objective of our study was to investigate the applicability and characteristics of CDSSs regarding chronic disease. The Web of Science, Scopus, OVID, and PubMed databases were searched using keywords from January 2000 to February 2023. The review was completed according to the Preferred Reporting Items for Systematic Reviews and Meta-Analyses checklist. Then, an analysis was done to determine the characteristics and applicability of CDSSs. The quality of the appraisal was assessed using the Mixed Methods Appraisal Tool checklist (MMAT). A systematic database search yielded 206 citations. Eventually, 38 articles from sixteen countries met the inclusion criteria and were accepted for final analysis. The main approaches of all studies can be classified into adherence to evidence-based medicine (84.2%), early and accurate diagnosis (81.6%), identifying high-risk patients (50%), preventing medical errors (47.4%), providing up-to-date information to healthcare providers (36.8%), providing patient care remotely (21.1%), and standardizing care (71.1%). The most common features among the knowledge-based CDSSs included providing guidance and advice for physicians (92.11%), generating patient-specific recommendations (84.21%), integrating into electronic medical records (60.53%), and using alerts or reminders (60.53%). Among thirteen different methods to translate the knowledge of evidence into machine-interpretable knowledge, 34.21% of studies utilized the rule-based logic technique while 26.32% of studies used rule-based decision tree modeling. For CDSS development and translating knowledge, diverse methods and techniques were applied. Therefore, the development of a standard framework for the development of knowledge-based decision support systems should be considered by informaticians.

## 1. Introduction

In the last decades, evidence-based medicine (EBM) has been regarded as a standard of patient care. New drug treatments, the results of controlled trials, and all new clinical findings are the main sources of evidence [1, 2]. Clinical decision-making should be made based on such evidence to achieve the best outcomes. EBM is widely applied in chronic disease management. Although it has many benefits, EBM implementation in clinical settings is not easy and rarely applied in clinical practices [3]. The most common barriers to applying EBM in routine care are how to access the latest evidence and the high volume of guidelines [4].

To achieve up-to-date evidence, clinicians must read a large volume of journals, articles, guidelines, and research outcomes daily. Because reviewing and memorizing them per day is a complex task for clinicians [5–7], clinical decision support systems (CDSSs) were developed to support clinicians to keep their knowledge up-to-date with the latest evidence in a smart way [8]. The CDSSs referred to any computerized systems that are used to support healthcare providers in the patient-related decision-making process to bridge a gap between clinical practice and evidence-based medicine [2]. Broadly, CDSSs are classified into two main categories to distinguish between their implementation and usage: knowledge-based and non-knowledge-based. Knowledge-based CDSSs use logical rules to produce outcomes in the form of recommendations to guide clinicians. There is always a source of knowledge in a knowledge-based CDSS, and rules are drawn from literature, patient-centered protocols, guidelines, or expert knowledge. Non-knowledge-based CDSS relies on machine learning or statistical pattern recognition techniques to simulate expert knowledge [2, 9].

Chronic diseases are recognized as the main cause of morbidity, mortality, and cost worldwide. Thus, controlling and preventing chronic diseases have become the main focus of health systems [10]. To achieve the best possible outcomes, evidence-based chronic disease prevention (EBCDP) emerged as a new approach to developing evidence-based programs to ensure that healthcare providers have access to up-to-date scientific evidence regarding chronic diseases [11]. Despite all efforts, there is a gap between knowledge generation and evidence implementation due to the inability of clinicians to search for and evaluate evidence [12]. There is a large volume of published studies describing the potential of knowledge-based CDSSs to increase evidence adherence in routine clinical practices [13–15]. In this regard, knowledge-based CDSSs have been developed to address the difficulty of implementing evidence-based medicine at the point-of-care in chronic disease [16]. Due to the popularity and applicability of knowledge-based CDSS and the complexity of chronic disease management, investigating developed CDSS programs in chronic diseases was the main focus of this study. Investigating the characteristics, applications, outcomes, and most favorable techniques of these informatics-based technologies are the main objectives of our study.

## 2. Method

In this systematic review, the Preferred Reporting Items for Systematic Review and Meta-Analysis (PRISMA) checklist was employed [17]. Published papers were retrieved from peer-reviewed journals from January 2000 to January 2023 based on search strategies in the following databases: PubMed, Ovid, Scopus, and Web of Science. Search strategies and extracted keywords were designed based on a formulated research question. Search strategies in each database are shown in Appendix A, Table A-1 in supplementary materials (available here). The PEO (population, exposure, and outcome(s)) approach was utilized to formulate the research question. In this study, population refers to patients suffering from chronic disease, exposure refers to using the CDSS system, and outcomes refer to the effectiveness, capabilities, and features of applied tools.

### 2.1. Study Selection and Extraction Criteria

The inclusion criteria were as follows: (1) Studies that included chronic diseases, (2) studies that involved physicians in clinical decision support systems through patient management, (3) studies that focused on disease management or health promotion, (4) only studies that developed or used knowledge-based CDSS and no other types of CDSS, and (5) target groups and users were clinicians. The exclusion criteria were as follows: (1) Studies that related to other domains except for health sciences, (2) studies that used only qualitative methods or simple usability tests, (3) studies that were published in a non-English paper, and (4) letter to the editor, reviews, and book chapters were not included in this study.

### 2.2. Data Extraction Process

Two reviewers independently screened titles, abstracts, and full-text of articles in a stepwise process based on the PRISMA checklist to find relevant studies for our review. A third reviewer was consulted and made the last decision in case of disagreement. Irrelevant studies were excluded based on exclusion and inclusion criteria. Required data and information were extracted from the eligible articles based on predefined categories. Hence, an electronic data sheet was designed for entering data. These predefined categories include a year of publication, main objective, study design, country, disease type, features, source of knowledge, method of knowledge translation, applied platform, users, and the main results. The gathered data were discussed by the authors.

### 2.3. Quality Appraisal

The Mixed Methods Appraisal Tool (MMAT) checklist was used for evaluating the appraisal of the eligible studies by two authors [18]. It contains five different specific questions for each study design to appraise articles. Each possible answer to each question comprises “Yes,” “No,” or “Cannot tell.” Total scores of “Yes” answers are calculated and assumed as the quality score for each study. Papers without a minimum quality score of three were excluded from this review.

## 3. Results

**Figure 1:**
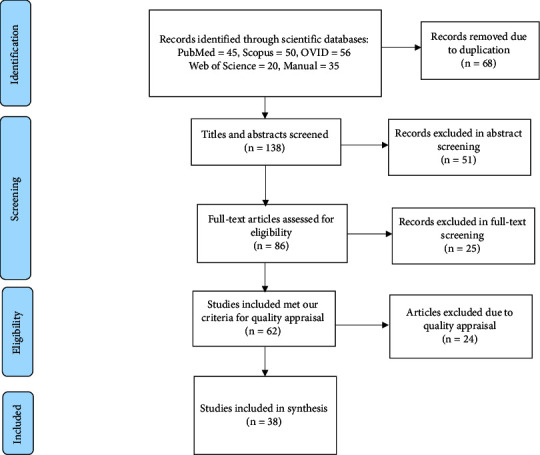
The flow diagram of the screening process based on the PRISMA method.

A systematic database search yielded 206 citations. The PRISMA flow diagram to describe the screening process is shown in Figure 1. It shows the process used to select the eligible studies that met the inclusion and exclusion criteria. In the first phase of abstract screening, 51 articles were excluded based on inclusion and exclusion criteria. After the full-text screening, 57 journal articles and four conference papers were identified as eligible studies. According to the MMAT checklist in the quality appraisal assessment phase, 24 studies were excluded due to low methodological quality. Thus, 38 studies were included in the synthesis and identified as eligible studies. The quality of 38 studies ranged from 60% to 100% which can be considered moderate to high. Of 38 articles, 11 articles scored between 60 and 70, 23 articles scored between 80 and 90, and 4 studies scored 100 based on the MMAT checklist.

Analysis of studies by year shows that the publication of studies has an almost upward trend. However, the rate of publication of articles in 2017 had the highest frequency with five studies.

Among 38 studies, 66.67% of them were descriptive (*n* = 25), 12.82% of them were cohort (*n* = 5), 10.26% of them were qualitative (*n* = 4), 2.56% of them were comparative studies (*n* = 1), and 7.69% of them applied mixed-methods research design (*n* = 3).

**Figure 2:**
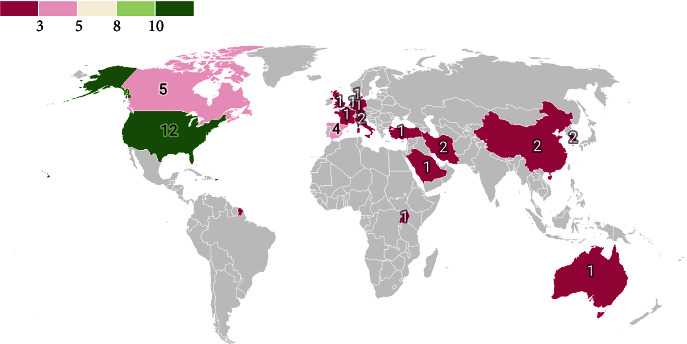
The distribution of studies on the map of the world.

In terms of country, 38 studies were retrieved from sixteen countries. Accordingly, 17 studies were devoted to the American continent (44.74%), 11 studies were conducted in European countries (28.95%), eight studies were conducted in Asian countries (21.05%), and one study (2.63%) was conducted in Africa and one in Australia. The distribution of studies by different countries is shown on the world map in Figure 2.

**Figure 3:**
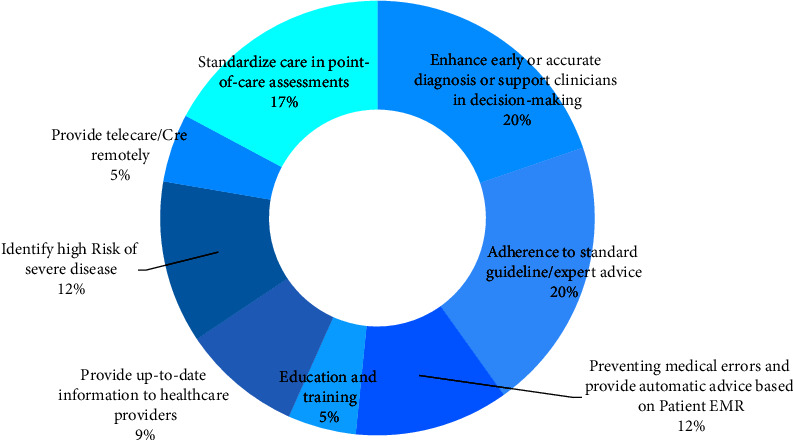
The frequency of the main approaches of developed knowledge-based CDSSs.

Though the main purpose of all CDSSs was to support healthcare providers in the decision-making process with embedded knowledge in the system and lead to high-quality care for patients, the main approaches of all investigated CDSSs can be divided into eight main categories. They include enhancing early or accurate diagnosis or supporting clinicians in decision-making, improving adherence to standard guideline/expert advice, preventing medical errors and providing automatic advice based on the patient's electronic medical record, educating and training, providing up-to-date information to healthcare providers, identifying patients at high risk of severe disease, providing medical care remotely, standardize the process of care at the point-of-care. The frequency and percentage of these six approaches are described in Figure 3. The details and characteristics of developed CDSSs in each study were described in Table 1.

### 3.1. The Application of Knowledge-Based CDSSs in Various Diseases

In medical sciences, all knowledge-based CDSS were developed to enhance patient care, diagnosis, follow-up, and routine care for a variety of diseases. Accordingly, 22 types of disease were investigated in this study. Asthma and cancer had the highest frequency among other diseases with a frequency of five articles (13.16%). Various chronic diseases were in the next category (10.53%). COPD, diabetes, chronic headache, pediatric disorders, and pharmaceutical consultations with two studies (6.25%) are other diseases considered in the reviewed articles. Other diseases include multiple sclerosis, chronic allergy, chronic kidney disease (CKD), chronic heart disease, hypertension, chronic pain management, pancreatitis, congenital heart disease (CHD), sleep disorder, COVID-9, eye disorders, osteoporosis, wound management, and thyroid nodules problems with one article (2.63%).

### 3.2. Methods to Convert Knowledge to Machine-Interpretable Formats in Developed CDSS

Since in this review, we focused only on knowledge-based CDSS, the source of knowledge was investigated. Based on the developed systems, the source of knowledge can be divided into four types of resources. They comprise standard guidelines which include 25 studies (65.79%), clinical protocols which include eight studies (21.05%), clinical rules extracted from patient data in combination with experts' knowledge which include two studies (5.25%), expert knowledge with two articles (5.26%), and massive evidence resources which include one study (2.63%).

Different methods and techniques were employed to translate the embedded knowledge into a machine-interpretable format. These various methods and their frequencies were categorized into 13 categories which are represented in Table 2. As it is apparent, 34.21% of studies utilized ruled-based logic techniques [21, 22, 25–28, 30, 31, 34, 37, 40, 41, 56] while 26.32% of studies used rule-based decision tree modeling [36, 42–44, 46, 47, 57] to convert the clinical knowledge into CDSSs in the form of computerized systems.

### 3.3. Software Development Methods to Develop Knowledge-Based CDSSs

After the embedded clinical knowledge was translated into the machine-interpretable format, nine software engineering methods were applied to implement these systems. However, three studies did not mention the applied techniques. In the following, we described the methods that were used in the reviewed articles:

1. User-centered design (UCD) approach with ten studies (26.32%): User-centered design or user-driven development is a framework in which users are involved at each stage of designing, developing, and developing a product, service, or process [58].
2. Iterative approach with seven studies (18.42%): Iterative designing methodology is a circular developing process that models, evaluates, and tests all of the developing stages of software [59, 60].
3. SOA with six studies (15.79%): Service-Oriented Architecture (SOA) is a kind of software designing methodology in which some services are considered through a communication protocol over a network [59].
4. MVC with three studies (7.89%): Model–view–controller (MVC) is a programming architecture that divides software into three separate parts [59].
5. BPM with two studies (5.26%): Business Process Modeling Notation (BPMN) is a software modeling method that models the various steps of developing software according to business process flow from end to end [60].
6. May's implementation theory with one study (2.63%): This theory provided a framework to index the domains and subcategories for a more thorough understanding of what worked and how it worked [61].
7. Prototyping with one study (5.26%): Prototyping refers to a model in which a prototype would be built from the desired product. It has key features of the final product under design but does not intend to show the main logic of the ultimate program [59, 60].
8. RAD approach with one study (5.26%): Rapid application development (RAD) is an agile project management strategy that is popular in software development [59, 60].
9. Knowledge-to-Action framework with two studies (5.26%): It is a conceptual framework intended to help those concerned with knowledge translation deliver sustainable, evidence-based interventions [62].
10. Workflow analysis with one study (3.13%): It is the process of breaking down the information flow of systems in the different workflow processes [63].

All of these applied techniques were developed on various platforms. The majority of studies (*n* = 30) were developed in the form of web-based software (78.95%), three CDSSs were developed in the form of mobile-based applications (13.16%), one CDSSs was implemented in windows-based software (2.63%), one of them was implemented as a simulated model (2.63%), and one CDSS was kind of web application which was accessible both in web-based form and in mobile-based application format (2.63%).

### 3.4. Features and Characteristics of Knowledge-Based CDSSs

**Figure 4:**
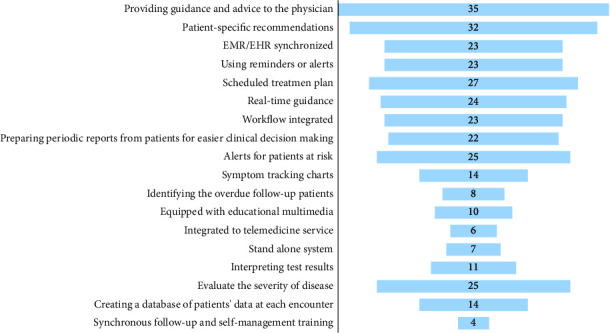
The features and capabilities of knowledge-based CDSSs.

The developed programs in the reviewed articles had different features and capabilities. The various features of these programs are summarized in Figure 4. Some of these features were common in developed CDSSs, and others were more specific to the purpose of the developed programs and the kind of disease. The most common features among the knowledge-based CDSSs included providing guidance and advice for physicians (92.11%), generating patient-specific recommendations (84.21%), integrating into electronic medical records (60.53%), and using alerts or reminders (60.53%). Self-management modules were only in programs integrated into telemedicine services (four studies).

### 3.5. Target Groups of Developed Systems

Knowledge-based decision support systems are generally developed to improve the quality of patient care, prevent unwanted medical errors, and provide timely decision-making. The users of developed systems comprise only physicians in 31 (81.15%) studies [14, 15, 19, 20, 22–24, 26–29, 32–34, 36, 39–44, 46, 47, 56], only nurses or team care members in two studies (5.26%) [21, 35], and both healthcare providers and patients in four studies (10.53%) [25, 30, 31, 37, 38, 45].

### 3.6. Effectiveness of Developed Clinical Decision Support Systems

We used the sign technique to investigate the effectiveness of developed CDSSs to improve decision-making in clinical processes by healthcare providers.

**Figure 5:**
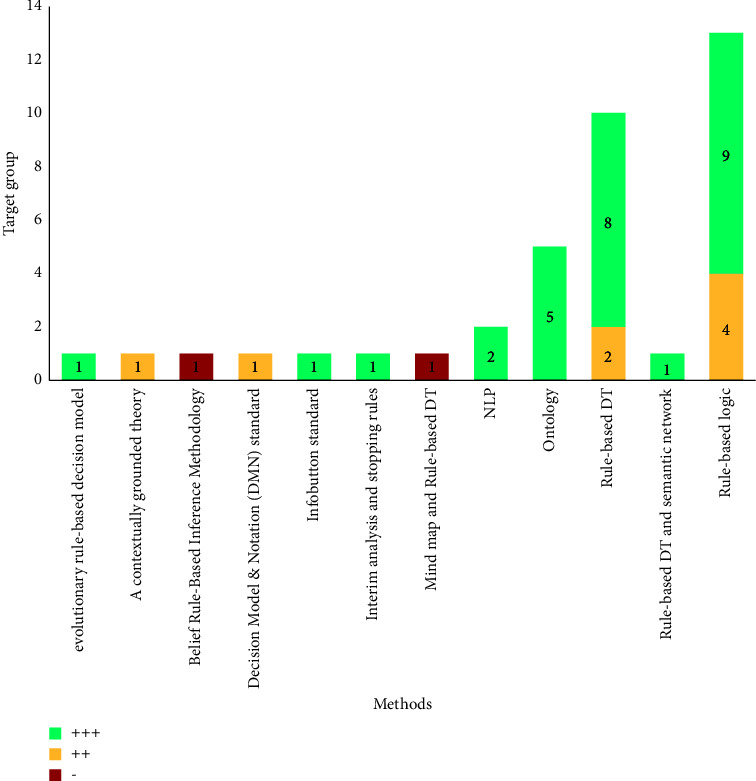
Effectiveness of applied methods based on their users.

According to analysis, about 73.68% of studies (*n* = 28) believed that developing CDSSs has a positive impact on the clinical decision-making process [14, 15, 20, 22, 24–30, 33–36, 38–45, 47, 53, 56] while about 21.05% of studies (*n* = 8) showed a positive impact to some extent [19, 21, 31, 37, 46]. Finally, only two studies (5.26%) declared that developing CDSSs was not effective in clinical practices [23, 32]. The effectiveness of applied techniques in reviewed CDSSs based on applied methods is described in Figure 5.

Different evaluation methods were employed in reviewed articles including qualitative methods [30, 33, 36–38, 41, 42, 46, 47], before-after study [14, 19, 23, 27, 33, 35, 38, 43, 44], the odd ratio [15, 28, 38], usability methods [19–21, 23, 24, 26, 28, 30, 37, 47], validation or performance evaluation [19, 20, 22, 24, 27–29, 39, 40, 42, 43, 56], simulation study [47], and think-aloud evaluation techniques [53].

Of 32 studies, only four studies did not employ standard evaluation methods [31, 40, 45, 46]. Accordingly, usability evaluation was conducted alongside the description of the CDSS development by applying different methods such as standard questionnaires (SUS, QUIS, etc.), and think-aloud methods [19–21, 23, 24, 26, 28, 30, 37, 47, 53].

To conduct the evaluation study, variant outcome measures or metrics were applied to the reviewed studies. All of these metrics can be classified into three main categories: system performance, clinical outcomes, and clinical processes. Metrics related to system performance included accuracy (13 studies), usability level (11 studies), qualitative methods (10 studies), ease of use (eight studies), sensitivity (seven studies), specificity (seven studies), odds ratio (six studies), ROC curve (three studies), NPV and PPV (two studies), error analysis (two studies), power estimation (two studies), alert fatigue (two studies), precision (one study), and resolution rate (one study).

Metrics related to clinical outcomes included clinical test enhancement such as Hb1AC level (13 studies), earlier diagnosis (four studies), and exacerbation rate of diseases such as emergency visits, oral steroid usage, and hospitalization (three studies).

Metrics related to clinical processes included overall process enhancement (12 studies), clinician feedback (13 studies), the rate of recorded clinical data before and after using the system (11 studies), system usage rate (eight studies), rate of completed tests and orders (seven studies), time of computation (six studies), system efficacy to enhance healthcare processes (six studies), new process acceptability (five studies), simulation-based assessment (four studies), and the number of visits (one study).

## 4. Discussion

According to the literature review, knowledge-based CDSS can improve clinical decision-making based on the rules embedded in the knowledge-based system and deducted rules based on an inference engine generally, while nonknowledge-based systems rely only on machine learning techniques as one of the artificial intelligence techniques to learn from stored data or historical experiences. In this review, we investigated the development of knowledge-based clinical decision support systems in chronic disease management regarding various characteristics and their applications. Our investigation showed that the main objective of all developed systems was to aid clinicians to make decisions with more confidence based on evidence. In this survey, knowledge-based CDSSs were considered computer-based and intelligent consultants.

However, various methods were employed to translate the clinical knowledge into machine-interpretable formats; logic rule-based algorithms; and rule-based decision tree techniques were the most widely used methods for developing knowledge-based clinical decision support systems in the reviewed articles. These two techniques are similar in terms of converting clinical protocols to decision rules. They have only differed in the representation of the extracted rules. In the decision tree approach, each branch represents a conditional statement (production rule) that can be easily understood by humans in addition to computer-based programs [27, 53]. These rules are generated using literature-based evidence, clinical protocols, paper-based guidelines, or patient-based evidence [8].

Knowledge-based CDSSs are built on two main components: knowledge-based and inference engines. In the knowledge-based database of CDSS, all of the embedded knowledge is available in the form of IF-Then rules [2, 14]. Next, the inference engine runs the built-in logic of evidence based on the combination of predefined production rules and entered patient data. The outputs of each CDSS contain alerts, diagnostic recommendations, probable risks, and treatment options [2].

Analysis of the most common features and capabilities in developed CDSSs showed that their functionality and applicability are diverse, including providing advice, alerts, patient-specific recommendation, real-time guidance, calculating the risk of disease, and training capabilities. Since the focus of this study is only on knowledge-based CDSSs, all of the investigated programs were equipped with generating advice for physicians and providing guidance through clinical decision-making [64].

Out of 38 systems, 23 of them are integrated with electronic health records (EHR) or electronic medical records (EMR). This capability can enhance the functionality and applicability of such systems to support clinicians with real-time guidance [2]. Sutton et al. believed that integrating CDSS with the EHR could provide them with advanced functions [2]. Another capability of knowledge-based CDSS was related to providing healthcare providers with a probable patient-specific diagnosis, treatment plan, or clinical advice based on the specific conditions of each patient. This feature allows physicians to easily have a wide range of possible diagnoses based on the available evidence instead of memorizing them. Such smart systems and integration CDSSs with other types of intelligent systems such as computerized provider order entry (CPOE) aid clinicians in moving toward patient-centered care and enhance health-related decisions [65].

Investigating the effectiveness of applying CDSSs in routine clinical practices revealed that most of them have a positive impact on clinical decision-making in terms of various aspects. However, the evaluation of digital health interventions is complex [66]. A wide range of metrics and indicators are applied in published studies like in reviewed studies. The represented metrics can be applied in further studies to measure the performance of developed CDSSs. The positive impact of developed CDSSs on clinical outcomes and performance showed that clinicians can benefit from developed CDSSs in various aspects.

**Figure 6:**
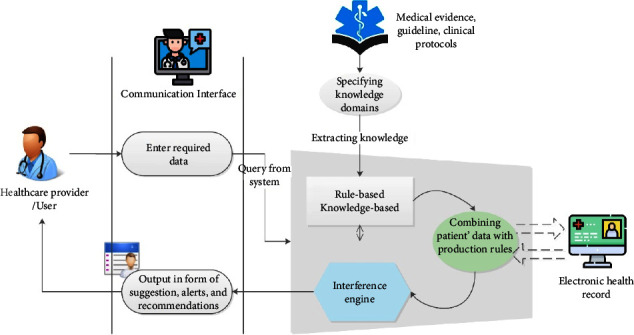
The general model of knowledge-based CDSS.

For CDSS development, various software development methods were applied, which were mentioned in the result section. Only two studies applied a specific framework for system design and implementation [46, 53]. Although the knowledge-to-action model is one of the most appropriate models for developing knowledge-based decision support systems [67], it has only been used in one study. Therefore, the development of a standard framework for developing knowledge-based decision support systems should be considered by informaticians. Summarizing the results, capabilities, and applied tools in the reviewed studies can lead to the generation of a general model for designing decision support systems in the chronic disease management domain. This model is shown in Figure 6.

Chronic diseases pose many challenges to health systems. More than 70% of the investigated systems achieved their goals successfully. Despite the fact that chronic diseases are broad, CDSSs have been developed in limited clinical domains. Evidence-based CDSSs for chronic diseases should be extended to different types of diseases to achieve the goal of the EBCDP approach. Managing patients with multimorbidity is a significant challenge for healthcare systems worldwide. Therefore, the development of decision support systems to manage multimorbid chronic diseases could be considered for further research in this field.

Since this study is the first attempt to review and analyze published articles regarding knowledge-based CDSSs in chronic diseases, it encounters some limitations. The results of some studies are published in the form of reports, letters to the editor, or other types of studies. Thus, we have not considered them based on our exclusion criteria. Also, some researchers may apply CDSS in routine clinical practices, but they have not published their attempts in the form of any research article or conference paper. It accounts for a publication bias. Thus, further research on specific domains in clinical practices may be done in the future.

## Figures and Tables

**Table 1:** The details and characteristics of developed CDSSs in reviewed articles.

#	Authors	Diseases	Platforms	Main approaches	Countries	Sources of knowledge	System developmental methods	Knowledge-based designing methods	Effectiveness or usefulness	Target groups	MMAT SCORE
1	Groccia et al. [19]	Headache	Web-based	To provide early diagnosis of headache	Italy	The National Institute for Health and Care Excellence (NICE)	Iterative approach along with service-oriented architecture	A contextually grounded theory	++	Patients and nurses	80
2	Muhindo et al. [20]	Pediatric	Mobile	This study aims to determine the adaptation, feasibility, acceptability, and sustainability of NoviGuide and its impact on nurse-midwives' knowledge in a rural hospital in eastern Uganda	Uganda	Uganda guidelines	User-centered design	Rule-based DT	+++	Physicians	80
3	Martinez-Garcia et al. [21]	Pediatric	Web application	To improve patients' healthcare, and patient safety by combining EBM into the decision-making in a routine of clinical practice	Spain	Different existing knowledge bases (e.g., massive reference bases, CPGs, systematic reviews, etc.)	Service-oriented architecture	Rule-based logic	++	Physicians	80
4	Paragomi et al. [22]	Pancreatitis	Web-based	Ariel dynamic acute pancreatitis tracker (ADAPT) is a digital tool to guide physicians in ordering standard tests, evaluating test results, and modeling progression using available data, propose emergent therapies	USA	A larger APPRENTICE dataset	Not mentioned	Rule-based logic	+++	Physicians	90
5	Mahmoud et al. [14]	Chronic disease	Web-based	A fully integrated electronic medical record system, with clinical decision support (CDS) systems to improve the quality of healthcare services in a primary care	Saudi Arabia	Clinical protocols	Not mentioned	Interim analysis and stopping rules	+++	Physicians	80
6	Cheung et al. [23]	Asthma	Web-based	CDSS integrated into the electronic medical record system in point-of-care for asthma to provide an asthma action plan for each patient	Canada	Canadian Thoracic Society Asthma Management Continuum	Prototyping	Mind map and rule-based DT	—	Physicians	90
7	Hosseini et al. [24]	Multiple sclerosis	Windows-based	To enhance the diagnosis of multiple sclerosis with the CDSS tool	Iran	Central nervous system diseases and MS diagnosis guidelines	MVC	NLP	+++	Physicians	60
8	Mammen et al. [25]	Asthma	Mobile	The intervention incorporated symptom monitoring by smartphone, smartphone telemedicine visits and self-management training with a nurse, and clinical decision-support software, which provided automated calculations of asthma severity, control, and step-wise therapy	USA	Guideline-based algorithms	May's implementation theory and iterative approach	Rule-based logic	+++	Physicians	90
9	Moja et al. [26]	Multispecialty	Web-based	A vendor-based multispecialty CDSS that generates patient-specific reminders based on high-quality point-of-care information services on clinical practice and quality of care in a general hospital	Italy	Swedish Finnish or SFINX	Not mentioned	Rule-based logic	+++	Physicians	80
10	Yu et al. [27]	Thyroid nodules	Web-based	The applicability of the knowledge-based CDSS to thyroid nodule treatment	Korea	American Thyroid Association and the 2016 CPGs of the Korean Thyroid Association	Iterative approach and business process modeling	Rule-based logic	+++	Physicians	90
11	Groenhof et al. [28]	Heart disease	Web-based	Modification of vascular risk factors is effective in reducing cardiovascular morbidity and mortality in patients with a cardiovascular condition	Netherlands	CVRM guideline	MVC	Rule-based logic	+++	Nurses	80
12	Ravikumar et al. [29]	Cervical cancer	Web-based	The focus of the current study deals with the evaluation of the precision of CDSS in a nonclinical setting	USA	American Society of Colposcopy and Cervical Pathology (ASCCP) guidelines	Workflow analysis	Rule-based DT	+++	Physicians	90
13	Cooley et al. [30]	Cancer	Web-based	The purpose of this project was to design and evaluate a simulated model of an algorithm-based CDS program for cancer symptoms	USA	Guideline	Mix-method user-centered design	Rule-based logic	+++	Physicians	90
14	Liu et al. [31]	Colorectal cancer	Web-based	It is possible to prevent or minimize certain CRC risks by adopting a healthy lifestyle	USA	Risk score calculation model	MVC	Rule-based logic	++	Physicians	80
15	Conway et al. [32]	Diabetes	Web-based	Automated clinical decision support systems (CDSS) are associated with improvements in healthcare delivery to those with long-term conditions, including diabetes	UK	NICE guideline	User-centered design	Belief rule-based inference methodology	—	Physicians	70
16	Otto et al. [33]	Allergy	Web-based	The FAST was designed to be implemented via functions already available within each practice's existing EMR and therefore likely already familiar to clinicians	USA	NIAID Guidelines for the Diagnosis and Management of Food Allergy	Rapid-cycle improvement	Evolutionary rule-based decision model	+++	Physicians	90
17	Hannon et al. [34]	Diabetes	Web-based	Compared screening for T2D among youth meeting ADA criteria between the intervention and control practices	USA	National guidelines	Not mentioned	Rule-based logic	+++	Physicians and patients	80
18	Yılmaz et al. [35]	Oncology	Web-based	To support the nurses' decision-making process about patients' needs	Turkey	Turkish version of NANDA-I, and nursing interventions evidence	User-centered design	Ontology	+++	Physicians	80
19	Séroussi et al. [36]	Hypertension	Web-based	To propose an innovative graphical interface to display medication recommendations as “therapeutic circles”	France	Guideline	Business process modeling	Rule-based DT	+++	Physicians	90
20	Blake et al. [37]	Sleep disorder	Web-based	Effective diagnosis of sleep disorders takes time and requires consideration of a lot of data	Australia	WHO guidelines	Iterative approach	Rule-based logic	++	Physicians and patients	90
21	Ennis et al. [15]	Chronic kidney disease (CKD)	Web-based	To facilitate adherence to guidelines, we were able to show an improved alignment between guideline recommendations and both test ordering and, in two instances, test results	USA	KDOQI guideline	User-centered design	NLP	+++	Physicians	80
22	Kuhn et al. [38]	Asthma	Web-based	To develop an electronic AAP decision support tool (eAAP) within the electronic health record (EHR) that could streamline the evidence-based NHLBI guidelines for providers	USA	NHLBI guideline	User-centered design	Ontology	+++	Physicians	90
23	Rahaman and Hossain [39]	Asthma	Web-based	The development and application of a BRBES to diagnose asthma based on signs and symptoms	Denmark	Datasets of 50 asthmatic patients	User-centered design	Ontology	+++	Physicians and patients	90
24	Woo et al. [40]	Chronic disease	Web-based	The current system aids decision-making by analyzing the correlation of prescription, diagnosis, and treatment with their results	Korea	National guidelines	Service-oriented architecture	Rule-based logic	+++	Physicians and patients	60
25	Dong et al. [41]	Headache	Web-based	To implement this CDSS in primary care units and community hospitals, to improve the level of diagnosis and treatment for headaches	China	ICHD guideline	Service-oriented architecture	Rule-based logic	+++	Physicians	60
26	Velickovski et al. [42]	COPD	Web-based	To manage chronic diseases with the application of integrated care pathways and the optimization and standardization of care processes	Spain	Free COPDKB knowledge database and GOLD guideline	Service-oriented architecture	Rule-based DT	+++	Physicians	60
27	Wagholikar et al. [43]	Cervical cancer	Web-based	To generate screening and surveillance recommendations for all female primary care patients in the institution,	USA	Guideline	User-centered design	Rule-based DT and semantic network	+++	Physicians	80
28	Jain et al. [44]	COPD	Web-based	To examine using an electronic CDS for detecting a1-antitrypsin (AAT)	USA	GOLD guideline	User-centered design	Rule-based DT	+++	Physicians	80
29	Liu et al. [45]	Chronic disease	Web-based	To deploy the system in one of the largest hospitals in China centers to manage type 2 diabetic patients	China	Guideline	Service-oriented architecture	Infobutton standard	+++	Physicians and healthcare providers	60
30	Kastner and Straus [46]	Osteoporosis	Mobile	To apply knowledge-to-action and the medical research council frameworks for complex interventions in the development of an osteoporosis clinical decision support tool	Canada	Canadian osteoporosis clinical practice guidelines	Knowledge-to-action framework and iterative approach	Rule-based DT	++	Physicians	100
31	Trafton et al. [47]	Pain management	Web-based	To develop and evaluate a CDSS that encourages the safe and effective use of opioid therapy for chronic, noncancer pain	USA	Evidenced-based automation (ATHENA)-Opioid therapy	Iterative approach	Rule-based DT	+++	Physicians	100
32	Calvo-Cidoncha et al. [48]	Pharmaceutical consultations	Web-based	To design and develop OntoPharma, an ontology-based CDSS to reduce medication prescribing errors. A secondary aim was to implement OntoPharma in a hospital setting	Spain	Nomenclator for prescription	Iterative approach	Ontology	+++	Physicians	70
33	Román-Villarán et al. [49]	Pharmaceutical consultations	Web-based	To design, develop, and validate an ontology-based CDSS that provides personalized recommendations related to drug prescription	Spain	CPG on the diagnosis and treatment of atrial fibrillation	Not mentioned	Ontology	+++	Physicians	70
34	Fraynal and Vogel [50]	Wound management	Model	They used the decision model and notation (DMN) standard as a knowledge representation format to implement a knowledge base for chronic wound material recommendation in phase-based therapy	Germany	“Moderne Wundversorgung,” from Kerstin Protz & Jan Hinnerk Timm	Not mentioned	Decision model and notation (DMN) standard	++	Physicians	60
35	Assadi et al. [51]	Congenital heart disease (CHD)	Web-based	This pilot study evaluates the effect of this CDSS on ED physicians' decision-making compared to usual care without clinical decision support	Canada	Defined scenario with experts	Prototype designing and iteratively designed	Rule-based logic	++	Physicians	70
36	Tanya et al. [52]	Eye disorders	Web-based	The objective was to standardize the triage and referral process while providing a more accurate provisional diagnosis and urgency	Canada	Expert knowledge	A cloud-based and UCD	Rule-based DT	+++	Physicians	60
37	Abtahi et al. [53, 54]	Asthma	Mobile-based	Proposing a framework for translating evidence into a guideline-based CDSS and developing an asthma-specific CDSS based on the proposed framework	Iran	GINA guideline	Knowledge-to-action framework and iterative approach	Rule-based DT	+++	Physicians	100
38	Chrimes [55]	COVID-19	Mobile-based chatbot	In the diagnosis and treatment of COVID-19, an effective clinical decision-support tool in form of a chatbot would ensure that best practices are followed	Canada	COVID guidelines and protocols	Agile method	Rule-based DT	++	Physicians	100

**Table 2:** Applied methods and techniques for knowledge translation.

Knowledge-basedelicitation methods	Count	Description
Rule-based logic algorithms	13 (34.21%)	A set of IF-THEN rules for knowledge statements was used to develop rule-based algorithms
Rule-based DT	10 (26.32%)	Rule-based techniques and classification methods were used to develop models
Ontology	5 (13.16%)	All of the knowledge was represented in the form of concepts and the relationships between concepts
NLP	2 (5.26%)	Converting parts of speech and written statements into a bag of words and weights gives computers the ability to understand the human language
Evolutionary rule-based decision model	1 (2.63%)	The evolutionary decision-making rule was found using attributes of patients extracted from similarity-based associative patient groups
A contextually grounded theory	1 (2.63%)	Grounded theory is introduced as an inductive, comparative methodology that provides systematic guidelines for gathering, synthesizing, analyzing, and conceptualizing qualitative data for theory construction
Belief rule-based inference methodology	1 (2.63%)	Belief rule-based expert systems (BRBESs) are widely used to capture uncertain knowledge and to accomplish the task of reasoning under uncertainty by employing belief rule-based and evidential reasoning
Info button standard	1 (2.63%)	An “infobutton” is a point-of-care information retrieval application or API that automatically generates and sends knowledge requests to online health knowledge
Interim analysis and stopping rules	1 (2.63%)	The term “interim analysis” is used to describe an evaluation of the current data from an ongoing trial, in which the primary research question is addressed, and which has the potential for modifying the conduct of the study
Mind map	1 (2.63%)	A mind map involves writing down a central theme and thinking of new and related ideas which radiate out from the center
Decision model and notation (DMN) standard	1 (2.63%)	It is a standard approach for describing and modeling repeatable decisions within organizations to ensure that decision models are interchangeable across organizations

## Data Availability

The study involves only a review of the literature without involving any data.

## Supplementary Materials

Supplementary Materials The search strategies in various databases are defined in Appendix.
