# Supplementary material for: The Application of Knowledge-Based Clinical Decision Support Systems to Enhance Adherence to Evidence-Based Medicine in Chronic Disease
Source: J Healthc Eng. 2023 May 29;2023:8550905. doi: 10.1155/2023/8550905 (PMC10241579; doi:10.1155/2023/8550905)
Supplement: Supplementary Materials — The search strategies in various databases are defined in Appendix. [file 8550905.f1.docx]

**Appendix A:**

Table A-1- Search strategies in each database

| **Database** | **Search strategies** | **Results (Count of studies)** |
| --- | --- | --- |
| PubMed | (("chronic disease"[Title/Abstract]) OR ("chronic diseases"[Title/Abstract]) OR ("chronic illness"[Title/Abstract]) OR ("Chronic Disease"[Mesh]) OR ("chronic"[Title/Abstract])) AND (("knowledge based"[Title/Abstract]) OR ("knowledge-based"[Title/Abstract]) OR ("rule-based"[Title/Abstract]) OR ("rule based"[Title/Abstract])) AND (("Clinical Decision Support Systems"[Title/Abstract]) OR ("Decision Support System"[Title/Abstract]) OR ("Clinical Decision Support"[Title/Abstract]) OR ("electronic Clinical Decision Support System"[Title/Abstract]) OR ("CDSS"[Title/Abstract]) OR ("Decision Support Systems, Clinical"[Mesh])) | 45 |
| Scopus | (TITLE-ABS-KEY ( "chronic disease" ) OR TITLE-ABS-KEY("chronic") OR TITLE-ABS-KEY("chronic illness")) AND ( TITLE-ABS-KEY ( "knowledge based" ) OR TITLE-ABS-KEY ( "knowledge-based" ) OR TITLE-ABS-KEY ( "rule-based" ) OR TITLE-ABS-KEY ( "rule based" ) ) AND ( TITLE-ABS-KEY ( "Clinical Decision Support Systems" ) OR TITLE-ABS-KEY ( "Clinical Decision Support System" ) OR TITLE-ABS-KEY ( "Clinical Decision Support" ) OR TITLE-ABS-KEY ( "CDSS" )) AND PUBYEAR > 2009 | 50 |
| Web of Sciences | (TS=("Clinical Decision Support Systems" OR "Clinical Decision Support System" OR "Decision Support System" OR "Clinical Decision Support" OR "electronic Clinical Decision Support System" OR "CDSS" )) AND (TS=("knowledge based" OR "knowledge-based" OR "rule-based" OR "rule based")) AND (TS=("chronic disease" OR "chronic diseases" OR "chronic illness" OR "chronic")) | 20 |
| OVID | (("chronic disease".mp. [mp=title, abstract, full text, caption text]) Or ("chronic".mp. [mp=title, abstract, full text, caption text]) or ("chronic illness".mp. [mp=title, abstract, full text, caption text])) AND (("knowledge based".mp. [mp=title, abstract, full text, caption text]) or ("Knowledge-based".mp. [mp=title, abstract, full text, caption text]) or ("rule-based".mp. [mp=title, abstract, full text, caption text]) or ("guideline-based".mp. [mp=title, abstract, full text, caption text])) to (yr="2010 -Current" and original articles) | 56 |
